# Current Advances in the Metabolomics Study on Lotus Seeds

**DOI:** 10.3389/fpls.2016.00891

**Published:** 2016-06-20

**Authors:** Mingzhi Zhu, Ting Liu, Mingquan Guo

**Affiliations:** ^1^Key Laboratory of Plant Germplasm Enhancement and Specialty Agriculture, Wuhan Botanical Garden – Chinese Academy of SciencesWuhan, China; ^2^Sino-Africa Joint Research Center – Chinese Academy of SciencesWuhan, China

**Keywords:** *Nelumbo nucifera*, lotus seeds, metabolomics, flavonoids, alkaloids

## Abstract

Lotus (*Nelumbo nucifera*), which is distributed widely throughout Asia, Australia and North America, is an aquatic perennial that has been cultivated for over 2,000 years. It is very stimulating that almost all parts of lotus have been consumed as vegetable as well as food, especially the seeds. Except for the nutritive values of lotus, there has been increasing interest in its potential as functional food due to its rich secondary metabolites, such as flavonoids and alkaloids. Not only have these metabolites greatly contributed to the biological process of lotus seeds, but also have been reported to possess multiple health-promoting effects, including antioxidant, anti-amnesic, anti-inflammatory, and anti-tumor activities. Thus, comprehensive metabolomic profiling of these metabolites is of key importance to help understand their biological activities, and other chemical biology features. In this context, this review will provide an update on the current technological platforms, and workflow associated with metabolomic studies on lotus seeds, as well as insights into the application of metabolomics for the improvement of food safety and quality, assisting breeding, and promotion of the study of metabolism and pharmacokinetics of lotus seeds; meanwhile it will also help explore new perspectives and outline future challenges in this fast-growing research subject.

## Introduction

Lotus (*Nelumbo nucifera*) is an aquatic plant, belonging to the mono-generic family Nelumbonaceae. As an important ornamental plant with beautiful flowers and pleasant fragrance, lotus has numerous common names (e.g., Chinese water lily, sacred lotus, and Indian lotus), and the lotus seed has been used as functional food for over 2,000 years in Asia (Guo, 2009). Lotus is an economically important aquatic vegetable in Asian countries, and China is the largest producer and consumer in the world, with a figure of 45,000 tons of dry seeds in an area of 0.5–0.7 million hectares of land per years (Guo, 2009). Lotus seed composes of three parts, namely seed epicarp, embryo, and cotyledons (Mukherjee et al., 2009). The lotus seed is popular as functional food due to its delicious taste, great nutritive and non-nutritive values (Mukherjee et al., 2009). After peeled and cored, lotus seed can be eaten raw, roasted, or ground and boiled into a syrup or paste (Moro et al., 2013). Lotus seed is also traditionally used to treat some ailments, including insomnia, palpitations, fever, poor digestion, chronic diarrhea, enteritis, tissue inflammation, and cancer. Lotus seed has been approved of use as “both food and medicine” by Ministry of Health of the People’s Republic of China (Zhang et al., 2015). The removed core of seed is seed embryo, and it is bitter, aromatic, and mainly consumed as medicinal practice or functional food. The lotus seed embryo has the properties of cleaning heat, cooling the blood, and it is traditionally used for the treatment of nervous disorders, insomnia, high fevers with restlessness, cardiovascular diseases such as hypertension and arrhythmia (Chen et al., 2007).

There have been a lot of studies investigating the purported health benefits of consuming lotus seeds, particularly over the last decade when people have generally become more aware of health issues and functional foods that may provide significant health benefits upon regular consumption (Fraser et al., 2014). Until now, lotus seed has been shown to possess several pharmacological properties, including antioxidant (Yen et al., 2006; Li et al., 2009; Xie et al., 2013; Liu et al., 2015), anti-amnesic (Jung et al., 2010, 2015), anti-inflammatory (Liao and Lin, 2011; Liao et al., 2011), and antitumor (Poornima et al., 2013, 2014) activities. It is generally believed that active components in lotus seed are responsible for these biological or pharmaceutical activities, and a wide variety of pharmaceutically active secondary metabolites have been found, including flavonoids and alkaloids (Chen et al., 2007, 2012a; Itoh et al., 2011). The comprehensive metabolomic profiling of these metabolites is of key importance to help understand their biological activities. Furthermore, the first draft of complete genome assembly of lotus has been obtained by Ming et al. (2013), and this opens up the post-genomic era of lotus. As an important part of suffix omics, metabolomics is powerful in assessing safety and quality of food, assistant breeding, metabolism and pharmacokinetics of lotus seeds derived products, and so forth (Oms-Oliu et al., 2013; Johanningsmeier et al., 2016). The wide application of metabolomics in lotus seeds has gained tremendous momentum in recent years (Itoh et al., 2011; Chen et al., 2012a; Li et al., 2014; Zhao et al., 2014; Hu et al., 2015; Jung et al., 2015; Peng et al., 2015).

In this context, this review will provide an update on the current technological platforms, and workflow associated with metabolomic studies of lotus seeds, as well as insights into the application of metabolomics for the improvement in food safety and quality, assisting breeding, and metabolism and pharmacokinetics regarding lotus seeds. Meanwhile, the future challenges in metabolomics study on lotus seeds were also outlined in this brief review.

## Metabolomics Study on the Lotus Seeds

### Metabolomics: An Overview

Metabolomics focuses on the analysis of the full set of endogenous or exogenous low molecular weight entities of approximately < 1500 Da (metabolites) in a biological system (cell, biofluid, tissue, organ, organism, etc.; Ibanez et al., 2013). Metabolites include a large number of molecules with diverse chemical and biological properties, which can be present in a broad range of concentrations, typically millimolar to femtomolar concentrations (Castro-Puyana and Herrero, 2013). Plant-derived food products are a rich source of diversified metabolites. In contrast to the essential primary metabolites (lipids, carbohydrates, organic acids, etc.) shared by animals and microorganisms, plant secondary metabolites have protective potential and health-promoting benefits that range from the stimulation of antioxidative mechanisms to a reduced risk of cancer incidence and cardiovascular disease (Schreiner et al., 2012). Previous studies have shown that lotus seeds are rich in flavonoids and alkaloids (Mukherjee et al., 2009; Duanmu et al., 2010; Li et al., 2014). Among them, flavonoids, a large group of almost 6000 secondary plant metabolites, are the most numerous of naturally occurring phenolic compounds, sharing a common carbon skeleton of two benzene rings (ring A and B), joined by a 3-carbon bridge (C_6_-C_3_-C_6_; Peluso et al., 2015). In recent year, flavonoids have received considerable attention because of their health benefits in the prevention of many chronic diseases, such as cardiovascular diseases, type II diabetes, neurodegenerative diseases, and various types of cancers (Visioli et al., 2011). As for alkaloids, they are another type of plant secondary metabolites, usually possess some predominately pharmacological activity, and in some cases, have medicinal or ecological use (Yang and Stockigt, 2010). The major alkaloids found in lotus seeds are bisbenzylisoquinoline alkaloids including liensinine, isoliensinine and neferine, and these alkaloids have various beneficial pharmacological effects, such as anti-depressant, anti-arrhythmia, anti-pulmonaryedema, and anti-HIV properties (Hu et al., 2015).

### Technological Platforms

From an analytical-chemistry perspective, lotus seeds can be considered complex solid matrices consisting of 100s of 1000s of compounds (nutrients and non-nutrients; Mukherjee et al., 2009). A big challenge in the metabolomic analysis of lotus seed is the incredible chemical diversity of the compounds. This means that there is no “one size fits all” approach to effectively analyze all of these compounds (Johanningsmeier et al., 2016). Different analytical strategies based on different platforms have been applied to metabolomics. These technologies include nuclear magnetic resonance (NMR), liquid chromatography–mass spectrometry (LC–MS), and gas chromatography–mass spectrometry (GC–MS). These techniques have their own advantage and disadvantage (Scalbert et al., 2014). NMR is a robust, and non-destructive analytical method. It is also a popular choice for metabolomic profiling due to the ability of high-throughput analysis (Singh and Roy, 2016). NMR requires minimal sample preparation, and can detect all organic classes. Signals on NMR spectra are proportional to the molar concentration of the compounds, thus the absolute concentration of the metabolites in the sample can be quantified (Johanningsmeier et al., 2016). The main limitation of NMR is its low sensitivity, which narrows its coverage of the food metabolome to predominant nutrients, such as sugars and amino acids. NMR is difficult to cover the non-nutrient metabolites occurring at low concentrations (Scalbert et al., 2014). The major applications of NMR in lotus seed metabolomics focused on the structure identification of pure compounds of alkaloids (Kato et al., 2015). GC–MS have an excellent separation capability, and a large pool of databases for metabolite identification. The major limitation of GC–MS requires compounds that are volatile or made volatile via chemical derivatization. GC–MS combined with chemical derivatization has been used to analyze constituents of the food metabolome such as mono- and disaccharides, sugar alcohols, organic acids, amino acids, and long-chain fatty acids (Uri et al., 2014; Warth et al., 2015). To overcome the main limitations for both NMR and GC–MS based analysis, LC–MS based methodology, characterized by its high separation capability, excellent sensitivity, high resolution, and wide detection dynamic range (Jorge et al., 2015), has become increasingly used to detect the secondary metabolites of lotus seeds, including flavonoids, alkaloids, and phenolic acids (Wu et al., 2013). LC–MS consists of two parts, LC system and MS system which are used for separation and detection of metabolites, respectively. Reversed-phase high performance liquid chromatography (HPLC) is doubtlessly the most widely used in metabolomics analysis with the excellent ability of separation. For a particular application, the chemical and physical properties of the column (e.g., particle size and column dimensions) need to be taken into account (Wu et al., 2013). For example, three HPLC elution columns (the Waters Sunfire C_18_, Xbrige Amide C_18_ and Atlantis^®^ T 3 columns) were compared, and the Atlantis^®^ T 3 column gave the best peak resolution for the analysis of flavonoids in lotus seed embryos (Chen et al., 2012a). In comparison to conventional HPLC, ultra-high performance liquid chromatography (UHPLC) begins to dominate as the LC method of choice (Gika et al., 2014) due to the much improved chromatographic resolution. Recently, UHPLC is gradually used in the metabolomic analysis of the lotus seeds (Lv et al., 2015; Peng et al., 2015), while HPLC was a main method used before (Chen et al., 2007, 2012a). Several modern chromatographic methods have also been developed for the analysis of metabolites in lotus seed. Wu et al. (2004) analyzed three alkaloids (liensinine, isoliensinine, and neferine) from lotus seed embryos by counter-current chromatography using two types of two-phase solvent systems. Duanmu et al. (2010) also analyzed three alkaloids from lotus seed embryos by high-speed counter-current chromatography method using pH-gradient elution mode. Zhu et al. (2011) firstly applied micellar electrokinetic chromatography to quantitatively analyze three alkaloids (liensinine, isoliensinine, and neferine) in lotus seeds embryos.

The most commonly used tandem mass spectrometers include triple quadrupole (QqQ), quadrupole-time of flight (Q-TOF), ion trap-time of flight (IT-TOF), linear trap quadrupole-Orbitrap (LTQ Orbitrap), quadrupole-Orbitrap (Q-Orbitrap). These mass analyzers can be divided into low-resolution mass spectrometry (LRMS) and high-resolution mass spectrometry (HRMS) by mass resolving power and mass accuracy. The typical resolving power of LRMS, such as QqQ and IT, are below 2000 FWHM (full width at half maximum) of the mass peak. TOF mass has the resolving power usually in the range of 12,000–50,000 FWHM, whereas the Orbitraps usually reach even higher values (up to 500,000 FWHM; Rubert et al., 2015). The resolving power of the MS instrument is crucial for the identification of unknown compounds, and the metabolomic study on plant-derived food has entered a rapid development period with the widespread applications of HRMS (Oms-Oliu et al., 2013). However, the metabolomic analysis of lotus seed selected LRMS as the major detection methods in the past, while the use of HRMS is far from satisfactory to date due to the expensive price and low penetration rate of HRMS (Lin et al., 2013; Zhou et al., 2013; Li et al., 2014; Peng et al., 2015). In addition, in combination with spectrum detector such as UV or DAD, LC–MS can provide additional on-line structural information for each individual peak in a chromatogram, which has contributed to the identification of metabolites in lotus seeds. For example, an unknown compound in lotus seed was tentatively identified as kaempferol or luteolin plus a rutinose by LC–MS, and the aglycone was subsequently confirmed as luteolin, based on the UV λ_max_ (Band II) at 255.2 nm (Li et al., 2014).

### Metabolomics Workflow for Lotus Seeds

To date, there are no standard guidelines described for every step of the work flow in metabolomic studies using LC–MS. However, metabolomic analysis using LC–MS usually consists of a sequence of steps including sample preparation, metabolite extraction, pre-analytical procedure, metabolite separation and detection, and data analysis (Cevallos-Cevallos et al., 2009). Solid samples in metabolomic analysis are usually be placed immediately in a liquid nitrogen tank after harvesting, and then stored at -20 or -80°C before use (Shepherd et al., 2015). However, some plant-derived foods, such as lotus seeds, tea, and coffee, could be stored at ambient temperature upon use, and there is no need to store at low temperature before analysis although the metabolites may be reflected by microorganisms or enzymes (Chen et al., 2012a; Lee et al., 2015). The extraction procedure is aimed at maximizing the amount and concentration of the metabolites of interest, and thus a critical step in metabolomics (Cevallos-Cevallos et al., 2009). The extraction procedure for targeted analysis relies on the previous knowledge of the analytes, such as solubility, polarity, and stability. There are also several other aspects to be considered for extraction, including extraction method, the solvent characteristics, the ratio of solvents and sample, duration of extraction and temperature. Undoubtedly, the most popular extraction method is solvent extraction (Kim and Verpoorte, 2010). Flavonoids and alkaloids are the major second metabolites in lotus seeds, and specific extraction procedure should be chosen for the targeted analysis of these metabolites (Duanmu et al., 2010; Li et al., 2014). Flavonoids are commonly extracted from plant-based food with methanol, ethanol, water or their combination, but in some cases these solvents are acidified (Naczk and Shahidi, 2004). Chen et al. (2012b) optimized the extraction protocol of lotus flavonoids through an orthogonal design, and showed that the solvent was the most important factor as compared with other factors like extraction time, and temperature. The highest yield of flavonoids was achieved with 70% methanol-water and a solvent: tissue ratio of 30:1 at 4°C for 36 h. The alkaloids in lotus seeds are usually extracted with aqueous methanol, aqueous ethanol, or acidified solvents because of their alkalinity (Wu et al., 2004; Chen et al., 2007; Zhu et al., 2011). However, novel methods for extraction of metabolites are seldom used in the analysis of lotus seed metabolomics (Kim and Verpoorte, 2010).

In all of the situations mentioned-above, the aim is to have a spectrum of targeted metabolites as broad as possible in the subsequent metabolomic analysis. However, the analysis of the targeted compounds may be disturbed by the complex matrices. In these cases, a further pre-concentration and purification of the targeted compounds from complex plant extracts is necessary. Solid-phase extraction (SPE) is a simple technique for such a treatment (Kim and Verpoorte, 2010). Sample fractionation and/or concentration can be activated by the solubility and functional group interactions among sample, solvent, and adsorbent. For example, the total flavonoids in lotus seed embryos were enriched and concentrated before HPLC analysis by an Oasis HLB SPE cartridge (Waters, USA) filled with reversed-phase polymeric sorbent (Chen et al., 2012a). The last step is data treatment which always involves the identification of the metabolites as a new start. Identification of the metabolites may be the most difficult step in metabolomic studies, especially when the data were collected by LRMS. Indeed, the results of any metabolomic analysis are biologically and chemically uninterpretable without metabolite identification. Usually, the metabolites can be unambiguously identified using analytical standards, or tentatively identified based on molecular weight, MS/MS data, oﬄine or online database matching (Oms-Oliu et al., 2013). The major secondary metabolites in lotus seed are flavonoids (Chen et al., 2012a) and alkaloids (Chen et al., 2007; Itoh et al., 2011). The fragmentation patterns of flavonoids, including the *O*- and *C*-glycosyl flavonoids, have been extensively studied using ESI-MS/MS. For flavonoid *C*-glycosides, the fragmentation pathway of *C*-glycoside at sugar moiety is difficult due to its resistance to hydrolysis of the C–C bond (Ferreres et al., 2011). The fragmentations in *C*-glycosides occur preferentially at the glycidic moiety, and the nature of the sugar is assigned by the loss sequence of hexose residues (-120 and -90 Da), the pentosyl residues (-90 and -60 Da), and the rhamnosyl residues (-104 and -74 Da). Compared with *C*-glycosides, the rupture of *O*-glycosyl flavonoids is easy and characterized by the loss of the sugar moiety (Ablajan et al., 2006; Liu et al., 2006). Liensinine and its analogs, isoliensinine and neferine are three major alkaloids in lotus seeds, which all belong to bisbenzylisoquinoline alkaloids (Chen et al., 2007). Zhou et al. (2007) elucidated the fragmentation patterns of these three alkaloids using ESI-MS/MS and hydrogen/deuterium (H/D) exchange. All these three alkaloids displayed major diagnostic fragments that formed by the cleavage of the C1′–C9′ bond resulting in positive group CD, and the loss of 4-ethyl-1-phenol or 4-ethyl-1-methoxybenzene following rearrangements. The relatively stable fragment ions formed by the elimination of H_2_O, CH_3_NH_2_, CH_3_OH, and CH_3_-N = CH_2_ could also be found in ESI-MS/MS spectra. Two new bisbenzylisoquinoline alkaloids, norisoliensinine and 6-hydroxynorisoliensinine were identified in lotus seed plumules by the above fragmentation pathways (Lin et al., 2013).

Another major challenge of metabolomics involves data processing and analysis. A typical metabolomics experiment requires large numbers of samples to generate results that are statistically rigorous, and effective software tools for rapid data mining procedures and alignment are essential to address the vast amount of data. After data processing, multivariate statistical techniques are utilized to analyze the huge data, and various multivariate methods, such as principal components analysis (PCA), principal components regression (PCR), partial least squares (PLSs), canonical discriminant analysis (CDA), feature weighting (FW), and cluster analysis (CLA) can be chosen (Oms-Oliu et al., 2013). For example, 17 main difference ingredients were identified from 64 samples of four different lotus organs, and PCA was used to differentiate the characteristic components of the four different lotus organs. All of the samples could be classified into four clusters, and no misclassification of these four groups occurred, which indicated that the samples in this organ were significantly different from those in other organs, with the majority of the difference ingredients being alkaloids and flavonoid glycosides (Zhou et al., 2013).

## The Application of Metabolomics in Lotus Seeds

### Metabolomics in Food Safety and Quality Control for Lotus Seeds

Food safety is a major concern for governments and people all over the world nowadays. As one of the factors affecting food safety, pesticide residues in foods and related commodities are of widespread concern (Castro-Puyana and Herrero, 2013). The main pests and diseases affecting the yield of lotus seeds are Rot, *Myzus persicae*, and *Prodenia litura*. However, the studies of pesticide residues in lotus seed are still in the beginning stages (Miao et al., 2013a,b). In order to control the pests and diseases, pesticides are widely used, especially organochlorine and pyrethroid pesticides which may result in high levels of pesticide residues in lotus seeds (Miao et al., 2013a). Miao et al. (2013a) analyzed 36 pesticide residues in 20 batches of lotus seeds by gas chromatography with electron-capture detection (GC-ECD). The results showed that the pesticide residues (decamethrin) were only found in two samples, and no pesticide residues were detected in other samples. And the levels of detected residues were below the permissible level ruled by the national standard of the People’s Republic of China. In addition, a modified quick, easy, cheap, efficient, rugged, and safe method (QuEChERS) coupled to GC-ECD based method was also introduced for rapid extraction of 36 pesticides in lotus seeds by optimizing extraction solvent (acetone, ethyl acetate, acetonitrile, *n*-hexane and *n*-hexane in combination with ethyl acetate) and purifying agent (neutral alumina, primary secondary amine, graphite carbon block and florisil). Two out of 24 batches of lotus seeds were found to be contaminated with *trans*-chlordane, although their levels were below limits of quantification (Miao et al., 2013b).

Another most important factor for consumer acceptance of foods is food quality, which is a complex parameter involving multiple aspects, such as food composition, food properties, flavor and aroma (Castro-Puyana and Herrero, 2013). And foods routinely undergo chemical, physical, and biochemical changes during pre-harvest and post-harvest time, throughout processing and storage, and over the course of their shelf life, right up until the time they are eaten by a consumer. These changes in food metabolites directly affect food quality, which has great implications for human health and well-being. Thus, a metabolomic approach can be quite powerful in expanding our understanding of the links between food composition and sensory quality (Pinu, 2015; Johanningsmeier et al., 2016). The contents of proteins, carbohydrates, unsaturated fatty acids, and minerals are traditionally the main indicators for the quality of lotus seeds (Zhang et al., 2015). However, lotus seeds are mainly used as functional foods in recent years, and the kinds and amounts of flavonoids (Chen et al., 2012a) and alkaloids (Chen et al., 2012a) are gradually the important indicators of the quality. Until now, 30 flavonoids and 10 alkaloids were identified in lotus seeds as shown in **Tables 1** and **2**, respectively, and their contents have been measured in various lotus seed samples. Chen et al. (2012a) identified five flavonoid-*O*-glycosides (rutin, hyperoside, isoquercitrin, kaempferol 3-*O*-robinobioside, isorhamnetin 3-*O*-rutinoside) from lotus seed embryos by HPLC/DAD/ESI-MS^n^, and then measured their contents by UV. The total content of flavonoids in lotus seed embryos was 730.95 mg 100 g^-1^ dry weight, and the contents of these five flavonoids were 407.90, 53.75, 63.78, 140.78, and 64.74 mg 100 g^-1^ dry weight, respectively. More recently, the flavonoids from nine lotus tissues in five stages of growth were identified and quantified by HPLC-DAD and HPLC-ESI-MS^n^. As a result, 38 flavonoids were identified in all samples. Most importantly, flavonoids in lotus seed plumules differed significantly from the other remaining tissues. Thirteen *C*-glycosyl flavonoids and six *O*-glycosyl flavonoids were identified in lotus seed plumules, in which 11 *C*-glycosyl flavonoids and five *O*-glycosyl flavonoids in these flavonoids were discovered for the first time in lotus (Li et al., 2014). The total flavonoids increased in seed plumules, decreased in seed coats and kernels, or remained constant in lotus seedpods as the tissues developed. In addition, it was reported that lotus seed epicarp was also rich in flavonoids (Chen et al., 2012a). It grows into black (full ripening stage) from green (green ripening stage) during ripening (Liu et al., 2015). Liu et al. (2015) investigated the polyphenols of lotus seed epicarp at different ripening stages by HPLC-ESI-MS/MS, and four polyphenol compounds were identified, including two flavan-3-ols (catechin and epicatechin) and two quercetin glucosides (hyperoside and isoquercitrin). The polyphenols contents of lotus seed epicarp at the green ripening stage, half ripening stage and full ripening stage are 13.08, 10.95, and 6.73%, respectively. The contents of flavan-3-ols decreased, and that of quercetin glucosides increased as the seed ripened. Liensinine, isoliensinine and neferine, which are the predominant alkaloids in lotus seeds, are another kind of biologically active ingredients in lotus seeds (Zhu et al., 2011; Lin et al., 2013). Zhu et al. (2011) applied micellar electrokinetic chromatography to quantitatively analyze the liensinine, isoliensinine, and neferine in different parts of lotus seeds embryos. The results showed that the contents of the three alkaloids in green leaves of lotus embryos are three to five times higher than that in white roots of lotus embryos. Furthermore, the content of liensinine is the indicator of qualification control for lotus seed embryos in *Pharmacopeia of People’s Republic of China*, and the content of liensinine should not be less than 0.20% (National Commission of Chinese Pharmacopoeia, 2010). The above data would provide valuable information for the quality control of lotus seed and its derived products.

**Table 1:** The flavonoids identified in lotus seeds.

No	Compounds	Reference
1	Rutin	Chen et al., 2012a
2	Hyperoside	Chen et al., 2012a
3	Isoquercitrin	Chen et al., 2012a
4	Kaempferol 3-*O*-robinobioside	Chen et al., 2012a
5	Isorhamnetin 3-*O*-rutinoside	Chen et al., 2012a
6	Isorhamnetin-3-neohesperidose	Li et al., 2014
7	Diosmetin-7-rutinose	Li et al., 2014
8	Quercetin-3-neohesperidose	Li et al., 2014
9	Luteolin-7-rutinose	Li et al., 2014
10	Myricetin 3-*O*-glucoside	Chen et al., 2012a
11	Myricetin 3-*O*-glucuronide	Chen et al., 2012a
12	Quercetin 3-*O*-glucuronide	Chen et al., 2012a
13	Astragalin	Chen et al., 2012a
14	Syringetin 3-*O*-glucoside	Chen et al., 2012a
15	Isorhamnetin 3-*O*-glucoside	Chen et al., 2012a
16	Kaempferol 3-*O*-glucuronide	Chen et al., 2012a
17	Quercetin	Kredy et al., 2010
18	Orientin	Li et al., 2014
19	Isoorientin	Li et al., 2014
20	Vitexin	Li et al., 2014
21	Isovitexin	Li et al., 2014
22	Apigenin-6,8-di-*C*-glucose	Li et al., 2014
23	Luteolin-6-*C*-glucose-8-*C*-pentose	Li et al., 2014
24	Luteolin-6-*C*-pentose-8-*C*-glucose	Li et al., 2014
25	Apigenin-6-*C*-glucose-8-*C*-xylose	Li et al., 2014
26	Apigenin-6-*C*-xylose-8-*C*-glucose	Li et al., 2014
27	Schaftoside	Li et al., 2014
28	Isoschaftoside	Li et al., 2014
29	Apigenin-6-*C*-glucose-8-*C*-rhamnose	Li et al., 2014
30	Apigenin-6-*C*-rhamnose-8-*C*-glucose	Li et al., 2014

**Table 2:** The alkaloids identified in lotus seeds.

No	Compounds	Reference
1	Liensinine	Wu et al., 2004
2	Neferine	Wu et al., 2004
3	Isoliensinine	Wu et al., 2004
4	1-(4′-hydroxybenzoyl)-6,7-dimethoxy-3,4-dihydroisoquinoline	Xu and Jian, 2008
5	Nelumboferine	Itoh et al., 2011
6	Nelumborines A	Itoh et al., 2011
7	Nelumborines B	Itoh et al., 2011
8	Higenamine 4′-*O*-β-D-glucoside	Kato et al., 2015
9	Norisoliensinine	Lin et al., 2013
10	6-Hydroxynorisoliensinine	Lin et al., 2013

### Metabolomics-Assisted Breeding of Lotus Seeds

Human selection and breeding of lotus seeds have been more than a 1000 years (Li et al., 2010). The breeding programs have been intensified in recent decades, and metabolomic strategy is gradually applied to facilitate strategic breeding efforts by biochemical phenotypes among lotus varieties (Lopez et al., 2015). To a large extent, the basic breeding goal for lotus is to cultivate improved cultivars resistant to cold, drought, and/or various diseases with high yield. Furthermore, as functional food, the contents of functional components in lotus seed are important indexes for the quality of lotus seeds. The breeding goal for lotus also includes cultivating the varieties of high contents of functional components. Chen et al. (2013) analyzed the flavonoids in 12 lotus cultivars by HPLC-DAD-ESI-MS^n^. The 12 cultivars were of the four genotype groups: rhizome lotus of *N. nucifera*, seed lotus of *N. nucifera*, flower lotus of *N. nucifera*, and *N. lutea*. Seed lotus, flower lotus, and rhizome lotus of *N. nucifera* are classified by the use and morphological differences (Guo, 2009). The results showed that the total flavonoid concentrations in lotus seed coats varied with germplasm, from 18.0 to 104.5 mg/100 g fresh weight. In addition, the functional components of 17 lotus local varieties from Korea, China, Vietnam, and Thailand were comparatively analyzed. The lotus seeds from Vietnam had the highest flavonoid content, which were 2.25 times more than the flavonoid content in samples with the lowest levels, that is, the seeds from China. Meanwhile, the lotus seeds from China had the highest alkaloid content, and contained 2.08 times more alkaloid content than the seeds from Korea, which had the lowest alkaloid levels in 17 lotus local varieties (Zhao et al., 2014). Metabolites have be used as a marker in breeding of lotus seeds, and desirable characteristics of high functional component contents at the germplasm level can be introduced into varieties by cross sexual hybridization (Fernie and Schauer, 2009).

### Targeted Metabolomics in the Metabolism and Pharmacokinetics Study on Lotus Seeds

The metabolism and pharmacokinetics of biologically active ingredients in lotus seeds focus on the three major alkaloids, liensinine, isoliensinine, and neferine (Yu et al., 2013; Hu et al., 2015). The metabolism of neferine in dog hepatic microsomal incubations was analyzed and characterized by HPLC-ESI-MS/MS. Six metabolites were identified as isoliensinine, liensinine and four novel bisbenzyltetrahydroisoquinoline alkaloids named as 6-*O*-desmethylneferine, 2′-*N*-desmethylneferine, 2′-*N*-6-*O*-didesmethylneferine, and 6,13-*O*-didesmethylneferine. All these metabolites were desmethyl or didesmethyl products of neferine, and *N*-demethylation and *O*-demethylation were two important metabolic pathways of neferine in dog hepatic microsomal incubations (Zhou et al., 2007). Yu et al. (2013) investigated the association of liensinine, neferine, and isoliensinine with eﬄux transporters. The transcellular transport study in Caco-2 cells, MDCK/MDCK-MDR1 and MDCK/MDCK-MRP2 cells demonstrated that liensinine, neferine, and isoliensinine are substrates of *P*-glycoprotein, whereas MDR-associated protein 2 is not involved in the transport process, suggesting that *P*-glycoprotein could be responsible for the absorption and distribution of the three alkaloids. The pharmacokinetics and metabolites of neferine in rat after a single oral administration were investigated by LC–MS. The plasma concentration-time curves of neferine (10, 20, and 50 mg/kg, i.g.) showed double absorption peaks with the first peak at 10 min and the second peak at 1 h. The t^β^_1/2_ of these three doses were 15.6, 22.9, and 35.5 h, respectively. Neferine distributed rapidly into different organ systems, with the highest concentrations found in the liver, followed by the lung, kidney and heart at doses of 10 or 20 mg/kg. At 50 mg/kg dose, concentrations of the kidney and lung were higher than those of others. Moreover, neferine was mainly metabolized to liensinine, isoliensinine, desmethyl-liensinine, and desmethyl-isoliensinine (Huang et al., 2007). The pharmacokinetics of the three alkaloids (liensinine, isoliensinine, and neferine) in rat plasma was further examined by UPLC–MS/MS. Multiple reaction monitoring (MRM), one of the targeted metabolomics methods in use, was used to monitor the transitions between the protonated molecules at *m*/*z* 611, 611, 625 [M+H]^+^ and the product ions at *m*/*z* 206, 192, 206 for the analysis of liensinine, isoliensinine, and neferine, respectively. The method was linear over the concentration range of 5–1000 ng/mL, and recoveries were more than 75.3%. The main pharmacokinetic parameters after intravenous administration included *t*_1/2_ (h), *V_d_* (L/kg), MRT (h), CL (L/h/kg), AUC_0_-_t_ (ng/mL⋅h) and AUC_0_-_∞_ (ng/mL⋅h; Hu et al., 2015).

## Conclusion and Outlook

Lotus, which is distributed widely throughout Asia, Australia and North America, is one of the oldest cultivated aquatic plants in the world. As an important functional food, lotus seeds are traditionally used for the treatment of nervous disorders, insomnia, high fevers with restlessness, poor digestion, chronic diarrhea, enteritis, tissue inflammation, cardiovascular diseases such as hypertension and arrhythmia (Chen et al., 2007; Mukherjee et al., 2009). A variety of pharmacological activities of lotus seeds to human health have been found, including antioxidant (Yen et al., 2006; Li et al., 2009; Xie et al., 2013; Liu et al., 2015), anti-amnesic (Jung et al., 2010, 2015), anti-inflammatory (Liao and Lin, 2011; Liao et al., 2011), and antitumor (Poornima et al., 2013, 2014) activities. The benefits of lotus seed to human health greatly promote the metabolomic study of lotus with an focus on the two major types of its second secondary metabolites, flavonoids, and alkaloids. This review highlights the workflow and state-of-the-art progress in the metabolomic studies on lotus seeds, as well as to provide insights into the application of metabolomics in improving food safety and quality of lotus seeds, assisting their breeding, promoting the study of metabolism and pharmacokinetics of their major active metabolites. Not only will further metabolomic studies on this subject help promote the food security and quality as well as breeding, but also aid in elucidating the mechanisms of many biological activities of its derived products. Meanwhile, there are tremendous challenges in metabolomics study on lotus seeds. Instrumentation improvements, especially HRMS, are needed to make the detection of multiple metabolites more robust, comprehensive and accurate; more spectral databases on lotus metabolites, especially for lotus seeds, need to be developed to fully characterize the chemical composition in lotus seeds; standards for metabolomic setups need further development to get the valid data of lotus seeds. With the increasingly wide use in both food and traditional medicines, chemical biology features of lotus seed should be further explored by metabolomics technologies, and the development and formulation of new products and new usages derived from lotus seeds are also expected to emerge in the years to come.

## Author Contributions

All authors listed, have made substantial, direct and intellectual contribution to the work, and approved it for publication.

## Conflict of Interest Statement

The authors declare that the research was conducted in the absence of any commercial or financial relationships that could be construed as a potential conflict of interest.
